# Meteorological Report
†Report of the Council of the British Meteorological Society. Read at the Annual Meeting, May 27th, 1856.


**Published:** 1857-04

**Authors:** 


### METEOROLOGICAL REPORT.†

This report contains, amongst other valuable information, a paper on "The weather in connexion with the wheat crop," by F. W. Doggett, Esq.; a report on the relative value of Schönbein and Moffat's Ozonometers, by Dr. H. Barker; and an admirable paper, by Dr. Moffat himself, "On the physical character of the urine, in relation to disease and atmospheric conditions." Dr. Moffat believes "that the physical characters of the urine are as variable as the wind." He concludes by remarking

---

† Report of the Council of the British Meteorological Society. Read at the Annual Meeting, May 27th, 1856.

"That there is a greater quantity of solids excreted by the kidneys during decreasing readings of the barometer and thermometer, than when the readings are increasing; that the quantity is greater during ozone than no-ozone periods; and with directions of the wind from south than from north points of the compass; and that it is greater during calms than when there are atmospheric currents. It also appears that the quantity of solids is greater with dry than with moist air, which may be owing to the cutaneous exosmose being increased by the moisture in the atmosphere. We observe, then, that warmth and moisture of the air diminish the quantity of solids in the urine; and it is worthy of remark, that Mr. Copland Hutchinson attributed the rarity of calculus among sailors to their sleeping between the lower decks, where the *temperature* and *moisture* of the air increased to that degree, that the place became a vapour bath. The smaller quantity of liquids and solids in the period of the day between 11 A.M. and 5 P.M. may be attributed to the free action of the skin while the body was exposed to the open air. It also appears that the quantity of solids is greater at the commencement of periods of decreasing readings than at the period of increasing readings; and it is with the former class of readings that the maximum of diseases takes place."

